# Residual malaria among migrant workers in Myanmar: why still persistent and how to eliminate it?

**DOI:** 10.1186/s12879-021-06839-5

**Published:** 2021-11-10

**Authors:** Myat Htut Nyunt, Khin Myo Aye, Shine Thura Naing, Aye Su Mon, Mi Mi Htwe, Su Mon Win, Wai Myat Thwe, Ni Ni Zaw, Myat Phone Kyaw, Aung Thi

**Affiliations:** 1 Department of Medical Research, Ministry of Health and Sports, Yangon, Republic of the Union of Myanmar; 2 Department of Public Health, National Malaria Control Programme, Ministry of Health and Sports, Nay Pyi Taw, Republic of the Union of Myanmar

**Keywords:** Malaria, Migrants, Malaria elimination, Myanmar

## Abstract

**Background:**

Residual malaria is probably an important source for the re-emergence of malaria infection in the elimination era. Assessment to identify the factors influencing residual malaria in high-risk groups is needed to develop evidence-based decisions by stakeholders and policymakers.

**Methods:**

This study was conducted to explore the factors influencing the residual malaria infection among migrant workers in two sentinel sites (endemic vs. pre-elimination areas) in Myanmar using the mixed-model method.

**Results:**

A total of 102 migrant respondents (65 in Bamauk and 37 in Shwegyin) were included for the quantitative assessment using pretested questionnaires during household visits. Although 87.3% of them had insecticidal bed nets (ITNs/LLINs), only 68.3% of the migrants in Bamauk and 57.9% in Shwegyin used it regularly. The use of any bed net was high (79.9% in Bamauk vs. 91.0% in Shwegyin). The mean LLINs in their families were 1.64 (95%CI: 1.48–1.81) in Bamauk and 2.89 (95%CI: 2.67–3.11) in Shwegyin. Most of them received no health information for malaria prevention within the last year and their knowledge about malaria was low. Their working nature was a challenge for control measures against malaria in migrants.

**Conclusion:**

The strategy for distributing LLINs and health promotion activities for mobile/migrant populations should be reviewed, and an appropriate action plan should be developed for the specific migrant group. Moreover, health promotion activities for behavior change communication should be strengthened in the migrant population in Myanmar.

## Background

Myanmar is now moving forward to malaria elimination by 2030 as the decreasing trend of reported cases within the last decades. Between 2005 and 2015, malaria incidence in Myanmar was decreased by 81%, malaria mortality by 94%, and in-patient admission rate by 87% [1, 2]. However, 291 of 330 townships were classified as malaria-endemic, reporting a total of 110,146 clinical cases of malaria and 21 malaria-attributable deaths in these endemic areas where 43.9 million people were living in 2016 [1].

Meanwhile, malaria elimination was initiated in Greater Mekong sub-region countries, and regional political commitment was done in May 2015 [3, 4]. In Myanmar, a national strategic plan for intensifying malaria control and accelerating progress toward malaria elimination 2016–2020 was formulated. Five regions/states were selected for the elimination of malaria in 2016. Another five regions/states by 2019 and these milestones were followed by the prevention from the reintroduction of malaria in eliminated areas [1, 5].

According to the National Strategic Plan for malaria elimination by 2030 in Myanmar, there were efforts on detection, protection, and providing access to diagnosis and treatment for high-risk groups especially in migrants and mobile populations. It includes the free distribution of the long-lasting insecticidal nets (LLINs), promotion of their behaviour change communications, and early and prompt treatment [1, 2, 5].

However, an outbreak of vivax infection in southern and northern Myanmar was reported in 2018 and 2019 [1]. Residual malaria may be an important source for reemerging malaria in the pre-elimination area. Residual malaria transmission is defined as all forms of transmission that can persist although strenuous efforts to eliminate the disease [6, 7]. Many factors contributing to the cause of residual malaria were documented [1, 6, 8] in which included parasite factors, human factors, and health care activities. Residual malaria in migrants challenges the malaria elimination target in remote areas in Myanmar. Out of 330 townships, 10 were responsible for 60% of malaria cases in Myanmar in 2018 [1]. These townships were located in the hard-to-reach areas and migrants were working there. Within the last decades, a significant number of malaria cases were detected among the migrants. The elimination will not be achieved unless the migrant population receives the protection measures and early diagnosis and prompt treatment [2, 3]. Assessment on residual malaria among the migrants in these townships is needed to support the evidence-based decision to stakeholders and policymakers [7].

## Methods

### Study design

A cross-sectional descriptive study with analytical components using quantitative and qualitative approaches was used to explore the factors influencing residual malaria among migrant workers in Myanmar.

### Study site and sampling procedures

**Fig. 1:**
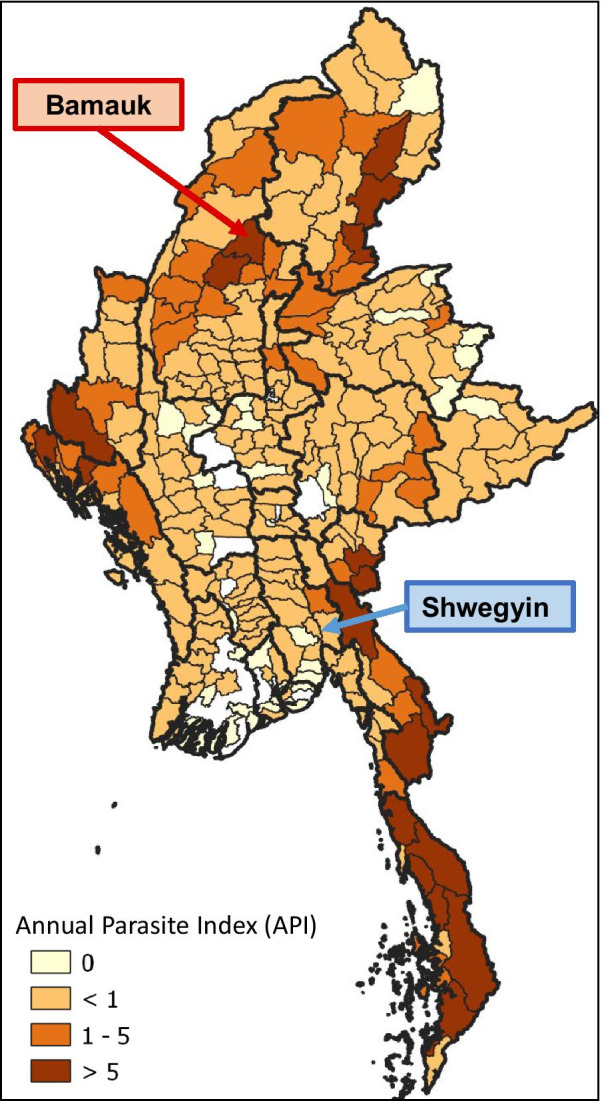
The study site related to the annual parasite index (API) in Myanmar as of 2019. Bamauk was an area of high malaria-reported cases, and Shwegyin was an elimination initiative township. The figure was adapted from the annual report of National Malaria Control Programme, 2019

Two townships were purposively selected for this study based on the number of malaria-reported cases, the presence of migrants, and feasibility. Shwegyin, Bago Region was a malaria-endemic area in the last decade, but few local cases were reported in 2017. The Shwegyin township was one of the malaria elimination initiatives township selected according to the WHO-recommended region-specific strategy in line with the principles of the global technical strategy for malaria 2016–2030[9]. Bamauk Township, Sagaing Region, was one of the malaria high burden areas in Myanmar (Fig. 1). As of 2018, Bamauk was included as one of the ten highest malaria prevalent townships in Myanmar. A total of 1608 cases were reported in Bamauk and 170 cases in Shwegyin in 2018. Although the reported malaria cases were different in these two townships, many migrants worked at the goldmines in both areas. Because of the nature of work, they lived in the deep forest and hard-to-reach receptive areas leading to increased exposure to malaria and poor access to routine health care services [10].

In each study site, half of the healthcare authorized areas of rural health care centers (RHCs) were randomly selected. For example, 3 out of 6 RHCs were randomly selected in Bamauk. Similarly, two of four RHCs in Shwegyin were randomly selected. At the RHC level, all migrants' housing was listed, and every alternate housing was selected systematically. During the household visit, only one individual who responded about the whole household was invited to participate in the study.

### Data collection

The quantitative data were collected during the household visit using pretested semi-structured questionnaires to explore the situation, prevention, and control of malaria among the migrants. The questionnaires were modified and adapted from the previous surveys conducted by the Malaria Consortium [11] and behaviour assessment survey [12]. The questionnaires were designated to collect information on the household listing, demography detail, nearest health facilities, bed net ownership and usage, malaria knowledge and recognition, and practice on malaria treatment-seeking behaviour. In each study site, three focus group discussions (FGD) and five key-informal interviews (KII) on local health authorities, community leaders, and migrant workers were conducted. The field survey-team that was composed of well-trained interviewers, collected data during household visits. The check-list was used to record the practice of the migrants in their household. The whole study was supervised by the principal investigators.

### Data management

All quantitative data were entered using the SPSS software package. All analyses used 95% CI, and p < 0.05 was accepted as significant. Qualitative data were transcribed and content-analyzed to identify patterns and themes and managed thematically by manually. The hand-coding and themes and sub-themes were identified based on the frequency of appearance. Discrepancies in coding were reviewed by the surveyors when necessary.

## Results

A total of 102 household visits composed of 65 migrant families in Bamauk and 37 in Shwegyin were conducted in this study. Most of them were working age group and male-dominant (Table 1). Very few migrants lived with families, while most were living with other migrants in the temporary camp situated in deep-forest in their workplace. The known history of malaria within five years was higher in Bamauk. Among them, about two-thirds of them agreed they have a risk of malaria (Table 1). However, their knowledge of malaria was not high. More than 75% of migrants know the disease "malaria," but only 43% of migrants in Bamauk and 57% in Shwegyin could mention the blood test was used for the diagnosis of malaria, while nearly 80% correctly mentioned the symptom of malaria. Although most of them recognized that mosquito bites can cause malaria, more than one-fourth have misconceptions about the causation of malaria (Table 2). Health staff was an important informer for the migrants in both sites; however, friends or neighbors were also a major source of information. Many migrants reported that they did not receive any information on malaria within the last 12 months (Table 2).

**Table 1:** Demographic characteristics of the study population

Characters	Bamauk n = 65 (%)	Shwegyin n = 37 (%)	Total n = 102 (%)
Age mean (SD)	34 (12.4)	38 (10.6)	36 (11.5)
Female Sex (%)	15 (23.1)	10 (27.0)	25 (24.5)
Number of household member Median (IQR)	1.5 (1.2.–5.5)	1.3 (1.7–6.9)	1.4 (1.5–6.1)
Known history of malaria within 5 years	23 (35.4)	6 (16.2)	29 (28.4)
Perceived risk of malaria	35 (53.8)	25 (67.6)	70 (68.6)

**Table 2:** Assessment of knowledge and source of information on malaria in two migrant populations

Category	Description	Bamauk n = 65 (%)	Shwegyin n = 37 (%)	Total n = 102 (%)
Knowledge on malaria	Know the disease, "malaria"	48 (73.8)	29 (78.4)	77 (75.5)
	Know the blood test for malaria diagnosis	28 (43.1)	21 (56.8)	49 (48.0)
	Know the symptoms of malaria	50 (76.9)	31 (83.8)	81 (79.4)
Knowledge on cause of malaria	Mosquitoes bite	51 (78.5)	32 (86.5)	83 (81.4)
	Drinking of dirty water	21 (32.3)	13 (35.1)	34 (33.3)
	Eating of some foods (e.g., banana)	18 (27.7)	9 (24.3)	27 (26.5)
	Living/visiting to forest	25 (38.5)	15(40.5)	40 (39.2)
	Sleeping in the forest	28 (43.1)	11 (29.7)	39 (38.2)
Knowledge on prevention of malaria	Bed net can prevent "malaria"	48 (73.8)	31 (83.8)	79 (77.5)
	ITN/LLIN can prevent the "malaria"	49 (75.4)	33 (89.2)	82 (80.4)
	Drinking of boiled water	25 (38.5)	19 (51.4)	44 (43.1)
	Do not know	11 (19.6)	4 (7.1)	15 (14.7)
Knowledge on anti-malaria	Know any antimalarials	15 (23.1)	11 (29.7)	26 (25.5)
	Artesunate	9 (13.8)	8 (21.6)	17 (16.7)
	Artemether	8 (12.3)	7 (18.9)	15 (14.7)
	Quinine	2 (3.1)	2 (5.4)	4 (3.9)
	Coartem	3 (4.6)	4 (10.8)	7 (6.9)
	Chloroquine	3 (4.6)	2 (5.4)	5 (4.9)
Source of information	Health staff	30 (46.2)	25 (67.6)	55 (53.9)
	Friends/neighbors	48 (73.8)	21 (56.8)	69 (67.6)
	Billboards	20 (30.8)	18(48.6)	38 (37.3)
	Poster	18 (27.7)	15 (40.5)	33 (32.4)
	Leaflet/Brochures	15 (23.1)	11 (29.7)	26 (25.5)
	TV/Radio	4 (6.2)	2 (5.4)	6 (5.9)
	Do not remember	25 (38.5)	21 (56.8)	46 (45.1)
Received information within the last 12 months	Yes	21 (32.3)	18 (48.6)	39 (38.2)
	No	32 (49.2)	11 (29.7)	43 (42.2)
	Not sure	12 (18.5)	8 (21.6)	20 (19.6)

**Fig. 2:**
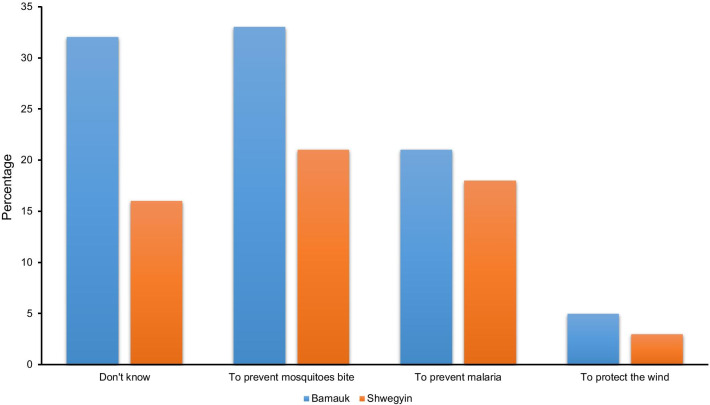
Knowledge on benefits of the use of LLINs among migrant populations in two study sites

Among the health facilities, the preferred choice by the respondent included drug sellers, quacks (non-registered medical practitioners), rural health centers, and private clinics. Simultaneously, community malaria volunteers (CMV) were not mentioned as their choice for health care, although CMVs were accessible in all nearby villages for diagnosis and treatment of malaria (Table 3). Although they had their bed net, less than one-third of Bamauk and about 40 percent in Shwegyin used it regularly. Moreover, bed net ownership and usage were better in Shwegyin compared to Bamauk. Interestingly, most of them were unable to mention the benefits of using ITNs/LLINs (Fig. 2). The reasons for the lack of bed net usage included too hot inside the net, dislike to use, and nature of their work. More than one-third of the families complained that their nets had holes or damage. Only half of them used the bed net last night before the household survey in both study sites (Table 3).

**Table 3:** Health-seeking behavior and behavior related to the prevention of malaria among the two migrant population

Category	Description	Bamauk n = 65 (%)	Shwegyin n = 37 (%)	Total N = 102 (%)
First choice of health facilities for their families	Township Hospital	1 (1.5)	1 (2.7)	2 (2.0)
	RHC	19 (29.2)	3 (8.1)	22 (21.6)
	Private clinic	5 (7.7)	15 (40.5)	20 (19.6)
	Village Health Workers	2 (3.1)	3 (8.1)	5 (4.9)
	Drug seller	20 (30.8)	10 (27.0)	30 (29.4)
	Quacks	18 (27.7)	5 (13.5)	23 (22.5)
Prevented measures from mosquito bite	Usage of LLIN	27 (41.5)	28 (75.7)	55 (53.9)
	Usage of non-treated net	25 (38.5)	18 (48.6)	43 (42.2)
	Mosquito coil	20 (30.8)	11 (29.7)	31 (30.4)
	Spray	1 (1.5)	1 (2.7)	2 (2.0)
	Cream/lotion	1 (1.5)	0	1 (1.0)
Ownership of the bed net	No. of bed nets in household, mean (95% CI)	1.07 (0.96–1.17)	1.33 (1.17–1.49)	1.21 (1.01–1.38)
	No. of LLIN in household, mean (95% CI)	1.64 (1.48–1.81)	2.89 (2.67–3.11)	1.81 (1.92–2.87)
	Having ITNs/LLINs	54 (84.4)	35 (94.6)	89 (87.3)
Usage of bed net	Regular	22 (33.8)	15 (40.5)	37 (36.3)
	Irregular	17 (26.2)	11 (29.7)	28 (27.5)
	Never use	11 (16.9)	5 (13.5)	16 (15.7)
Reasons for not using the net	Don't like	18 (27.7)	12 (32.4)	30 (29.4)
	Too hot inside	15 (23.1)	11 (29.7)	26 (25.5)
	Because of the nature of work	10 (15.4)	5 (13.5)	15 (14.7)
	Presence of holes/damage of the currently using net	25 (38.5)	17 (45.9)	42 (41.2)
	Presence of extra-/unused bed net	5 (7.7)	7 (10.8)	12 (11.8)
Use of the bed net last night	Yes	35 (53.8)	19 (51.4)	54 (52.9)
	No	30 (46.2)	18 (48.6)	48 (47.1)

Only 33.8% of the migrants in Bamauk and 40.5% in Shwegyin used the Long-lasting Insecticidal treated bed nets (LLINs) regularly. However, the usage of any bed net was high in both sites (79.9% in Bamauk vs. 91.0% in Shwegyin). More than 80% (85.4% in Bamauk and 88.9% in Shwegyin) said that they did not receive any health education session or activity to prevent malaria within the last two years. Only five fever cases were reported within two weeks before the survey (3 cases in Bamauk and 2 in Shwegyin). All five fever patients tried to recover by themselves, taking drugs from grocery shops. Among them, only one case in Bamauk was not recovered by self-treatment, and falciparum malaria was later diagnosed and treated by a private clinic in the nearby town.

**Fig. 3:**
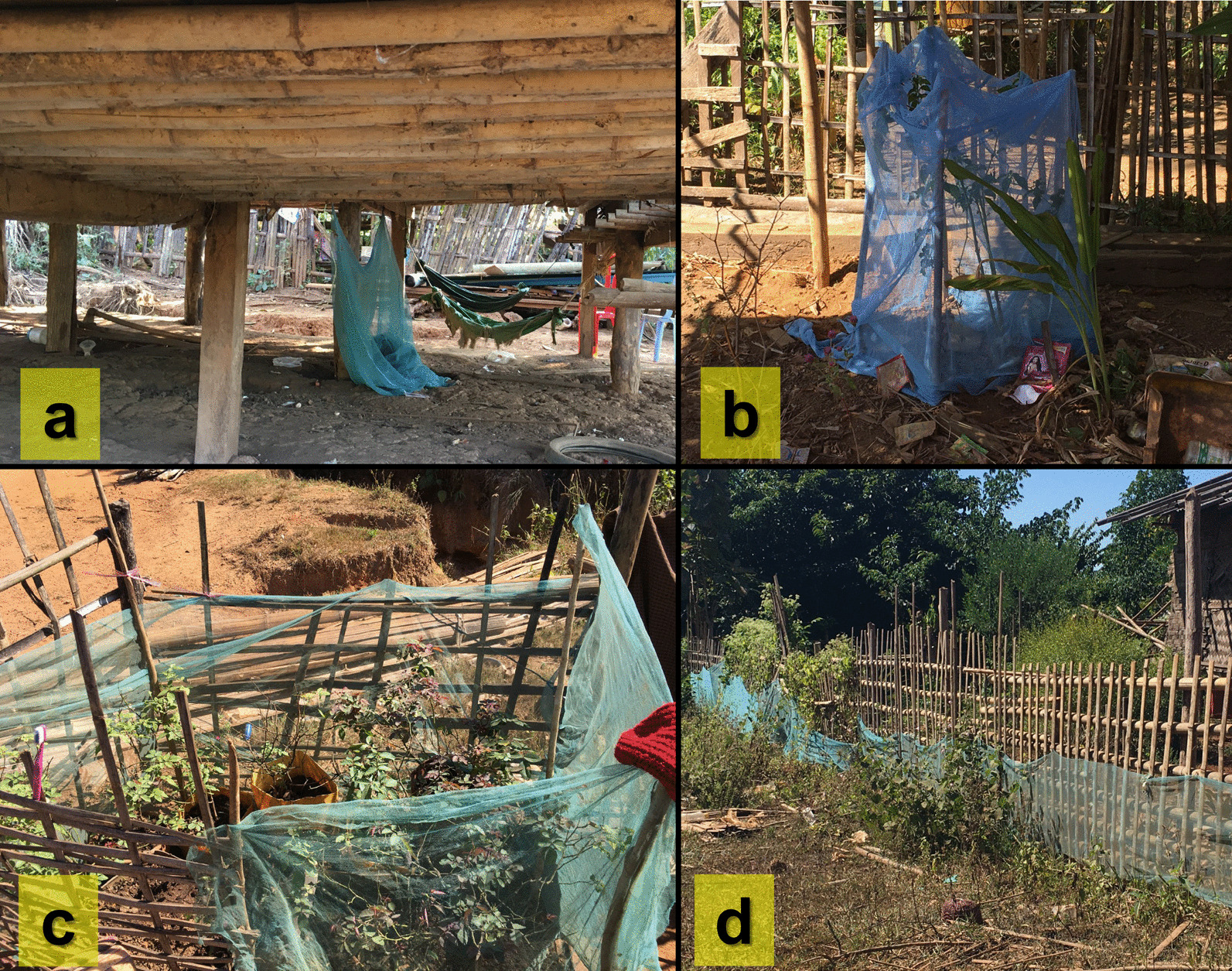
Misuses of the LLINs. **a** LLINs under the housing for animals **b**, **c** LLINs for plant cover **d** fence of the housing covered by LLINs. The figures were captured by the study team during the household visits

During household visits, misuse of the LLINs was observed in which included misuse of the LLINs for animal farms, plant covers, and fences of housing in four households in Bamauk and five households in Shwegyin (Fig. 3).

### Qualitative findings

One of the migrant workers in goldmine indicated that malaria knowledge gained from their previous native areas was as follows,

"We know that the bite of mosquitoes causes malaria, and it can be prevented by insecticidal bed net. But I got this message while I was staying in my native town, not here. In this migrant site, no health care person comes and gives information about malaria."

Simultaneously a migrant also pointed out that health promotion activities were not covered enough in their areas,

"In my previous place, there are many posters and billboards on the side of the road. Here, I found nothing. It would be better to have a similar billboard or poster at our place so that we can know the information."

One local health staff mentioned the lack of specific support for health promotion activities and behavior change communications.

"There is no specific budget allocation for behavior change communication (BCC) in this area. We have an old vinyl poster presented at the front of the hospital. We need specific support for information, education, and communication (IEC) materials to improve the BCC of the migrants."

Some migrants did not receive the free distributed LLIN, and a migrant woman expressed the opinion as follows,

"We got no insecticide-treated net from local health centers here, but we have the bed nets that were carried from our previous place."

The local community leader explained the reason for the lack of distribution to all migrants and the challenges of the health-related activities in their places as.

"Migrants are moving from one place to another place without prior notice. Moreover, some are working 48 hours continuously in the underground mine, followed by 48-hour rest. So, it is difficult to meet and give proper health education."

Moreover, one worker said his experience and reasons for lack of the usage of LLINs as;

"We have received the nets, but we don't know what it is exactly. So, we used it at night, and become a red rash appeared on the face and hand. We were afraid to use the net. Then, we do not use it until now."

## Discussion

Myanmar is moving forward for malaria elimination by 2030. To achieve this target, a malaria foci investigation to contain and eliminate locally transmitted cases is conducted according to the malaria elimination plan [5]. Meanwhile, drug resistance and residual malaria become challenges to the elimination target. Residual malaria in high-risk groups, especially migrants, leads to sporadic cases and outbreaks in controlled or pre-elimination areas. Unlike the residence, the migrant/mobile populations did not stay in one specific area for a long period. Because of the working conditions, the migrants were unable to use the bed net regularly during their sleeping time [13]. Furthermore, their worksites were located in the hard-to-reach areas where routine health care facilities were not easily accessible. Therefore, migrant workers were recognized as the high prioritized group for malaria [14].

In this study, the two study sites, malaria-controlled areas (Bamauk) and pre-elimination areas (Shwegyin) were included to explore the prevention of malaria among migrant workers. Although a few local cases were reported in Shwegyin in the last few years; known history of malaria within five years was common among migrants working in malaria-controlled areas. The Bamauk showed a high percentage of the known history of malaria within five years that coincided with the malaria cases reported [1].

Although many migrants in this study agreed they had a perceived risk for malaria, their general knowledge of malaria was not high. It was lower than the previous study conducted in the southern-Myanmar areas in 2013 [12]. This study highlighted that there were misconceptions on the cause, prevention, and control of malaria reflecting the inadequacy of health literacy. Most of them were not received health promotion activities within the last year. Qualitative findings confirmed that their knowledge of malaria was received from previous resident areas, not at the current workplace.

The reasons for the lack of providing health promotion activities were their migrant working nature, and routine health care activities were unable to reach all migrants at one time. Therefore, situation analysis of migrants in certain areas should be done before the control activities. The specific measures that would be suitable for a particular migrant site are needed to be conducted.

Most of them had at least a bed net; they did not use it regularly. This may be due to the nature of work and their behaviour [13]. Moreover, some misuses of the distributed LLINs were noted, similar to the previous findings [12, 15, 16]. It reflects their attitude and practice in the prevention of malaria by using LLINs. The provision of LLINs and preventative measures by upgrading the health literacy of malaria in migrants is important as residual malaria may pose significant challenges to achieving elimination goals in the migrant population [17].

The migrants are prone to get malaria in hard-to-reach areas where access to routine healthcare facilities was limited. This study highlighted that many migrants preferred the drug shop as their first choice for health care. Moreover, self-treatment for fever cases was common, and it was higher than the previous study [12]. The community malaria volunteers at the nearby village were not commonly selected to seek treatment for fever. Improper management of febrile cases in migrants may cause residual malaria and outbreak potential.

Lack of adequate knowledge on malaria prevention, including the benefits of using LLINs, leads to the improper use of the bed net, and lack of awareness on prevention of malaria were major challenges to eliminating malaria in the migrant population. The distribution of LLINs in migrants should be reviewed as the current distribution was not accessible by some migrants in this study.

Furthermore, health promotion activities, including the distribution of IEC (information, education, and communication) materials were not common in migrants although these were widely available in the urban and semiurban areas Unlike the residents, routine health promotion activities were not feasible to all migrants in one time and their working nature and available time should be concerned to provide the activities A specific strategy for migrant population by using all available channels for early diagnosis, prompt treatment, and prevention of malaria should be conducted. Moreover, surveillance of malaria among migrants should be strengthened.

As a limitation, the study did not focus on the behavior related to malaria prevention among migrants and residents in the study areas. As their health-seeking behavior and malaria prevention were recorded, as they mentioned, recall bias might affect the findings.

## Conclusion

Malaria infection among migrants is a hidden threat to achieving the elimination target. Therefore, a specific strategy focusing on eliminating and preventing residual malaria in the migrants should be emphasized in Sagaing and Bago Region in Myanmar.

## Data Availability

The datasets used and/or analysed during the current study are available from the corresponding author on reasonable request.

## Abbreviations

BCC: Behaviour change communication
CI: Confidence interval
FGD: Focus group discussion
IEC: Information, education and communication
LLINs: Long lasting insecticidal nets
